# Low-Carbohydrate Tolerant LAB Strains Identified from Rumen Fluid: Investigation of Probiotic Activity and Legume Silage Fermentation

**DOI:** 10.3390/microorganisms8071044

**Published:** 2020-07-14

**Authors:** Palaniselvam Kuppusamy, Dahye Kim, Ilavenil Soundharrajan, Hyung Soo Park, Jeong Sung Jung, Seung Hak Yang, Ki Choon Choi

**Affiliations:** 1 Grassland and Forages Division, National Institute of Animal Science, Rural Development Administration, Cheonan 31000, Korea; kpalaselvam@korea.kr (P.K.); ilavenil@korea.kr (I.S.); anpark69@korea.kr (H.S.P.); jjs3873@korea.kr (J.S.J.); y64h@korea.kr (S.H.Y.); 2 Faculty of Biotechnology, College of Applied Life Science, Jeju National University, Jeju 63243, Korea; pioioiq10@gmail.com

**Keywords:** *Lactobacilli*, minimal carbohydrate, silage, alfalfa, fermentation, organic acids

## Abstract

The objective of this study was to isolate and characterize lactic acid bacteria (LAB) with low carbohydrate tolerance from rumen fluid and to elucidate their probiotic properties and the quality of fermentation of *Medicago sativa* L. and *Trifolium incarnatum* L. silage in vitro. We isolated 39 LAB strains and screened for growth in MRS broth and a low-carbohydrate supplemented medium; among them, two strains, *Lactiplantibacillus plantarum* (*Lactobacillus plantarum)* RJ1 and *Pediococcus pentosaceus* S22, were able to grow faster in the low-carbohydrate medium. Both strains have promising probiotic characteristics including antagonistic activity against *P. aeruginosa, E. coli, S. aureus,* and *E. faecalis*; the ability to survive in simulated gastric-intestinal fluid; tolerance to bile salts; and proteolytic activity. Furthermore, an in vitro silage fermentation study revealed that alfalfa and crimson clover silage inoculated with RJ1 and S22 showed significantly decreased pH and an increased LAB population at the end of fermentation. Also, the highest lactic acid production was noted (*p* < 0.05) in LAB-inoculated silage vs. non-inoculated legume silage at high moisture. Overall, the data suggest that RJ1 and S22 could be effective strains for fermentation of legume silage.

## 1. Introduction

Lactic acid bacteria (LAB) are a heterogeneous group of Gram-positive microorganisms that have drawn attention for their probiotic potential, functionality, safety, and stability in various harsh environments. LAB strains could improve the quality of fermented foods (yogurt, kimchi, soy sauce, sauerkraut, fish and meat products, pickles, etc.) by improving the taste and health-promoting benefits of the product [1,2]. Certain attributes of probiotic organisms, including bile salt tolerance, survival in gastric and intestinal fluid, adhesion to epithelial cells, and probiotic stability, are considered vital [3]. Also, inclusion of LAB in foodstuffs increases the nutritional quality and storage life of fermented products and beverages by controlling food contamination and improving antimicrobial properties. LAB strains have been isolated from different foods and natural sources, including fermented foods, dairy products, sugar cane plants, soil, poultry farms, cattle feces, and fish waste. However, LABs isolated from the mammalian gut are more relevant to activity in animals than LABs that are obtained from other sources. The gastrointestinal microflora have diverse functions in animal production, and a greater quantity of LAB exists in the alimentary tract of ruminants than anywhere else [4]. Recently, LAB isolated from animal sources, such as rumen fluid, feces, and forages, has been shown to improve silage fermentation. These isolates have high mass, fast growth rates, good adhesion activities, and a high survival percentage in acidic and alkaline environments [5]. Silage additives are mainly divided into four categories: common fermentation stimulants, product inhibitors, nutrients, and aerobic deterioration inhibitors. The most suitable fermentation stimulants are bacterial inoculants; these can strongly increase the productivity of higher organic acids and increase the aerobic stability of silage. LAB can be classified into two major groups, such as homo-fermentative and hetero-fermentative LAB [6]. *Lactiplantibacillus plantarum* (also known as *Lactobacillus plantarum*) and *Pediococcus pentosaceus* are important homo-fermentative starter cultures used in the food and beverage industry to enhance the quality of products and restrict the growth of harmful microbes. The advantage of these LAB inoculants is that they do not adversely affect animal health, productivity, or safety [7,8].

Legumes commonly have thick fibrous stems that become less digestible as the crop matures. LAB strains are used as a silage inoculant to help fermentation of legume stems and improve the feed value for livestock [9]. Alternatively, legumes may be left to wilt in the field, then immediately prior to ensiling, suitable LAB additives can be applied during the silage harvesting process to reduce the negative effects of wilting [10,11]. Alfalfa is an essential forage crop because of its high palatability, nutritional value, wintertime hardiness, and drought tolerance [12]. Alfalfa silage is often difficult to ensilage because of its high crude protein concentration, large buffering capacity for acidic conditions, and low water-soluble carbohydrate (WSC) concentration; nevertheless, compared with alfalfa hay, alfalfa silage is naturally more effective because of reduced leaf losses when ensiling whole alfalfa plants [13]. Hence, alfalfa silage is considered to be a high-quality feed resource for livestock ruminants and non-ruminants. Chemical additives lend more stability than do biological additives, but biological additives, mainly *Lactobacilli*, improve the feeding value and stability of alfalfa silage [14]. Crimson clover is a legume fodder that also provides good-quality pasture, hay, and silage. Recently, Broderick et al. (2008) [15] reported that lactating dairy cows can use N from red clover more efficiently than that from other legume sources, as revealed by a lower urea concentration in milk and blood and less N elimination without decreasing milk yield and body weight. Based on that, we investigated the new low-carbohydrate-tolerant LAB strains for legume silage fermentation under high moisture conditions for prolonged storage. Also, we investigated the efficiency of the strains in controlling spoilage pathogens in silage in terms of their potent in vitro antibacterial, antifungal, antioxidant, and probiotic properties. 

## 2. Materials and Methods

### 2.1. Rumen Fluid for LAB Isolation

Rumen fluid was collected from Hanwoo steer cattle from a livestock animal farm located in the National Institute of Animal Science (NIAS), Cheonan-si, Korea. The rumen sample collection method was agreed by the Veterinary Division, NIAS. The rumen fluid was collected from a rumen-fistulated Korean Hanwoo steer cattle aged between 4–5 years. This steer was fed a diet composed of Italian ryegrass (IRG) silage with concentrate feed at the NIAS farm. The sample volume of rumen fluid was 50 mL collected through the fistula at 11 a.m. (after morning grazing), and the solid-phase was filtered through four layers of gauze and maintained anaerobically in an Erlenmeyer glass flask for 30 min at 37 °C. The collected rumen fluid was carefully shipped to the laboratory at 4 °C for further analysis [16].

### 2.2. Isolation of LAB Cultures

One milliliter of fresh rumen fluid was added to 9 mL autoclaved deionized H_2_O, and then a serial dilution was prepared (10^1^–10^5^) with sterile ddH_2_O. Meanwhile, 100 µL of each dilution was evenly spread on an MRS agar plate (Pronadisa, Conda, Spain) and kept at 30 ± 5 °C for 2 days. LAB colonies were grown on MRS agar plates and were isolated based on precise physical characteristics, including shape, size, color, and rough and/or smooth surface. Then, single colonies were selected and their growth patterns were analyzed in MRS broth under micro-aerobic conditions at 30 ± 5 °C for 48 h. In the end, two rapid-growth LAB strains were selected from the 39 isolates (RJ1 and S22). Furthermore, stocks of the LAB cultures were prepared using 80% glycerol (1:1) and were stored at −80 °C for further analysis. 

### 2.3. LAB Growth Pattern Analysis in Minimal Carbohydrate Medium

The isolated LAB cultures were screened by growth pattern analysis on a low-carbohydrate medium. The minimal carbohydrate medium consisted of 1 g K_2_HPO_4_, 1 g KH_2_PO_4_, 5 g yeast extract, 0.001 g FeSO_4_, 0.25 g MgSO_4_, 0.005 g NaCl, and 1 g glucose (Sigma, Gangnam-gu, Seoul, Korea) in 1 L distilled water, with pH adjusted to 7.0 [17]. This medium mimics the low-sugar environment for LAB growth that we used the growth profile analysis. The growth patterns of the LAB strains in the low-carbohydrate medium were analyzed at different time intervals using a microplate reader at 600 nm (iMark microplate reader, Bio-Rad, Tokyo, Japan).

### 2.4. Molecular Characterization by 16S-rRNA Gene Sequencing

The selected LAB strains were molecular characterized by 16S ribosomal RNA gene sequencing analysis. Total DNA was extracted from the LAB strains using a Qiagen DNA mini prep kit (Qiagen Korea Ltd., Jung-Gu, Seoul, Korea) according to the manufacturer’s guidelines. A purified DNA sample was sent to Bioneer Corporation, Korea, for 16S-rRNA gene amplification and sequencing. The obtained 16S-rRNA gene sequences were compared to those of known *Lactobacillus* strains in the NCBI-BLAST database, and the authenticity of the strains was confirmed. 

### 2.5. Biochemical Characterization of Isoalted LAB

Carbohydrate fermentation (API 50 CHL, BioMérieux, France) and extracellular enzyme production (API ZYM, BioMerieux, France) of the isolates were determined using the respective kits. Antibiotic susceptibility of the selected LABs was also tested (Yi et al. 2016) [18].

### 2.6. Antibacterial Assay of Isolates

The antibacterial activity of the LAB strains (RJ1, S22) was analyzed against various pathogenic bacteria using an agar-well dilution assay. Briefly, the LAB cultures were cultivated overnight and then centrifuged at 5000 rpm for 15 min at 4 °C. The centrifuged cell-free supernatants were collected separately and filtered by a 0.2 µm membrane filter. The filtrate was used as the extracellular fluid. Then, the bacterial pellets were suspended with a cell lysis buffer (Sigma-Aldrich, Saint Louis, MO, USA) and were sonicated. The lysed cell pellet was filtered and freeze-dried. After that, the supernatants and freeze-dried cell pellets were dissolved in saline and cell density was adjusted to an OD of 0.5, 1, and 2 at 600 nm. Aliquots (50 µL) of each type of pathogenic bacteria (*E. coli, P. aeruginosa, E. faecalis,* and *S. aureus*) were spread onto nutrient agar plates, 100 µL of LAB intra cellular fluid (ICF) and extra cellular fluid (ECF) were added to the appropriate wells, and the plates were left for 48 h in an incubator at 37 °C. At the end of the experimental period, clear inhibition zones were observed in the skim-milk agar plates [19]. 

### 2.7. Antifingal Activity of LAB Isolates by Agar Spot Method

The antifungal activity of the LAB isolates against fungal strains responsible for silage spoilage was analyzed by the method of Arasu et al. (2013) [20]. In brief, 25 mL of sterilized MRS agar medium was poured into Petri dishes. First, 10 µL of LAB strains RJ1 and S22 (OD 0.5, 1, and 2) were spotted on MRS plates; the plates were left at 35 °C for 24 h to allow bacterial growth. Next, 10 mL of sterile potato dextrose agar (10%) was mixed with 50 µL of fungal spore suspension and slowly transferred onto the MRS agar medium on the same Petri plates. The MRS plates were incubated for 72 h in an incubator at 37 °C. After incubation, LAB antagonist activity was measured by observing the clear zones of inhibition on the MRS agar plates. Control experiments were prepared with fungal spores alone. All experiments were done in biological triplicate (*n* = 3).

### 2.8. Antioxidant Activity of Isolates 

Aliquots (6.25, 12.5, 25, 50, and 100 µL) of overnight LAB cultures were added to 1 mL of an ethanolic DPPH solution (0.05 mM), the solutions were mixed by vortexing, and then were incubated at 37 °C for 30 min. The blanks contained ethanol alone, and DPPH solution alone and vitamin C were used as the negative control and positive control, respectively. After incubation for 30 mins, optical density of the LAB sample mixed with the DPPH solution was measured at 517 nm [21]. The percentage scavenging ability of LAB samples was calculated according to the following equation.

DPPH free radical (%) = [1−(X_sample_ − X_blank_)/ X_control_)] × 100.

### 2.9. Probiotic Characterization of LAB Isolates

#### 2.9.1. Bile Salt Tolerance Assay

The bile salt tolerance assay for LAB cultures was done as described by Ding et al. (2017) [22]. Concisely, the selected LAB was resuspended in saline at a concentration of 10^7^ CFU/mL. Then, the LAB suspension (1% *v/v*) was inoculated into an MRS broth comprising 0.3% oxgall. After different time intervals (0, 2, and 4 h) at 37 °C, we monitored the number of viable bacteria on the MRS broth with oxgall bile salt (0.3%) by measuring the OD at 620 nm using a SpectraMax i3x Microplate Reader (Molecular Devices, Seoul, Korea). The control consisted of MRS broth without bile salt. All experiments were repeated twice with duplicate analysis.

#### 2.9.2. Simulated Gastric and Intestinal Fluid Tolerance Tests

The simulated gastrointestinal fluid resistance of the isolated strains was assessed by a previously described method [23]. First, simulated gastric fluid was prepared as follows: 0.35 g of pepsin was dissolved in 100 mL sterile PBS, the solution was mixed well, and then the pH range was adjusted to 2.5–3.0 with HCl (1 M). 

For the simulated intestinal-fluid (SIF) tolerance test, 0.1 g trypsin was dissolved in 100 mL sterile PBS containing 1.2% sodium thioglycolate, 2.2% NaHCO3, and 0.44% sodium chloride, and the pH range was adjusted to 8–8.5 with NaOH (1 M). The freshly prepared solution was vortexed for 10 s and used for further analysis. To analyze the tolerance of the LAB cultures in simulated gastrointestinal fluid, 1% bacterial cultures were added with 5 mL of simulated gastric fluid and the mixture was incubated at 37 °C for 4 h. After incubation in artificial gastric fluid, the viability of the strains was calculated by the MRS agar plate-count method. The sample at 0 h was used as a control. Then, 0.5 mL of gastric fluid was added to 4.5 mL of simulated intestinal fluid (SIF), and the mixture was again incubated at 37 °C for different times (0, 24, and 48 h). After incubation, simulated gastrointestinal fluid dilutions were prepared (10^1^–10^3^ serial dilution) and cells in each dilution were plated on MRS agar (*n* = 3); then the plates were kept at 37 °C for 24 h and viable colonies (CFU/mL) were calculated. 

#### 2.9.3. Cell Surface Hydrophobicity, Auto-Aggregation, and Co-Aggregation Properties of LAB Strains

The cell surface hydrophobicity of the LAB strains was determined by the “bacterial adherence to hydrocarbons” method as described by Prabhurajeshwar et al. (2017) [24] and Li et al. (2020) [25]. 

The auto-aggregation and co-aggregation assays were performed according to the method of Ilavenil et al. (2016) [26].

### 2.10. Proteolytic Activity of LAB Cultures

The proteolytic activity of isolates was detected using the skim milk agar hydrolysis test [27] with slight modifications. The LAB culture (10 µL) with a cell density of 10^7^ CFU/mL was spotted on a skim milk agar plate containing 1% (*w/v*) skim milk and was incubated at 37 °C for 48 h. The proteolytic activity of the LAB strains was shown by the existence of a clear hydrolysis zone around the culture spot. 

### 2.11. In Vitro Legume Silage Production

#### 2.11.1. Legumes Silage Preparation

In this study, two legumes, alfalfa and crimson clover, were harvested from an experimental farm (NIAS, Cheonan, Korea) in the early budding stage. The collected legume crops were carefully transported to the laboratory in order to reduce the moisture level of the legume crops left in the sun to dry. When the moisture content of the legume crops reached 60–65%, the samples were cut into small pieces (~2 cm in size) using a mechanical cutter. After cutting, 100 g of each legume silage sample was added separately to the LAB additives (RJ1 and S22) at a concentration of 10^5^ CFU/g. The non-treated group received an equal volume of PBS alone. Then, the treated and control legume silage sample bags (10 × 15 cm) were quickly compressed by a vacuum sealer instrument (Food Saver, vacuum sealer machine, Seoul, Korea). Each group contained three replicates, and the silage sample bags were stored at room temperature. The fermentation period lasted for 180 days. After fermentation, the silage samples were kept in ice boxes during transportation and analyzed for the different microbial and biochemical parameters of legume silages [28].

#### 2.11.2. pH Analysis of Fermented Silage

We dissolved 10 g of each legume silage sample in 90 mL of autoclaved distilled water. The solution was mixed thoroughly on a shaker at 120 rpm for 60 min, and then the pH of the solution was tested using an inoLab pH meter (Xylem analytics, Weilheim, Germany).

#### 2.11.3. Total Microbial Population Analysis in Legume Silage

After pH measurement, the prepared aqueous extracts of the legume silage samples were used to quantify the microbial populations. The samples were serially diluted and total cell populations were detected using the Quantum Tx microbial cell counting method (Logos Biosystems, Seoul, Korea), as previously published [29]. Total cell staining dye is a fluorescent dye that permeates the microbial cell membrane of both live and dead cells. A 10 µL silage sample was mixed with each 1 µL cell staining dye and staining enhancer, and was kept in incubation for 30 mins. Then, 8 µL of cell loading buffer-I was added gently and the mixture of 5 µL was loaded into cell counting slides. The slides were centrifuged at 300 RCF for 10 min, and the sample was counted with the Quantum Tx microbial cell counter at light intensity level 5. In addition, mold and yeast were identified by the petrifilm kit method. About 1 mL of sample was poured onto the film, the film was incubated at 25 °C for 96 h, and then the number of colonies on the film sheet was counted.

#### 2.11.4. Organic Acid Analysis Using HPLC-RID

About 10 g of each legume silage sample was mixed with 90 mL of distilled water and shaken well at 150 rpm for 60 min in a rotary shaker (Vision shaking incubator, Gyeonggi-do, Korea). Then, the silage sample was acidified with 1 M hydrochloric acid (1:3, *w/w*) and the homogenate was centrifuged at 5000 rpm for 10 min. Next, 5 mL of the supernatant was filtered through a 0.22 μm filter. The filtrate was analyzed with an HPLC-RID detector (Agilent 1200, San Diego, CA, USA) to quantify the organic acid percentage in the silage sample. Chromatographic separation was done on an Aminex HPX-87H organic acid column (Bio-Rad Laboratories, Inc., CA, USA) fitted with a stationary phase. The isocratic mode of the mobile phase was assessed using 0.05 M H2SO4. The mobile-phase solvents were filtered and degassed before analysis. The flow rate was set to 0.7 mL/min, and the column temperature was 30 °C. The sample injection volume was 20 µL. Organic acids were detected at an ultraviolet (UV) detector wavelength of 210 nm. Organic acid ranges were calculated by comparing standard peak areas with the unknown peak area.

### 2.12. Statistical Analysis

Statistical data are expressed as mean ± standard deviation (SD); the analysis was done using MS Excel 2007. The statistical significance of differences between means was compared by one-way analysis of variance (ANOVA) using Duncan’s test using SPSS 16.0 software (IBM SPSS software, Armonk, NY, USA). Each sample was tested in triplicate (*n* = 3), and significance was considered at *p* < 0.05.

## 3. Results 

### 3.1. Isolation and Selection of LAB from Rumen Fluid

Here, we isolated 39 LAB strains from rumen fluid. The isolated strains were first subjected to growth pattern analysis in MRS broth; among them, four strains (RJ1, S22, 42, and R22) showed rapid growth in the MRS broth. Based on the OD_590_ value and the pH ranges in the growth medium, two strains (RJ1 and S22) showed high growth rates and had the ability to quickly decrease pH compared to the other isolates (data not included).

### 3.2. Growth Pattern Analysis in Low-Carbohydrate Medium

**Figure 1:**
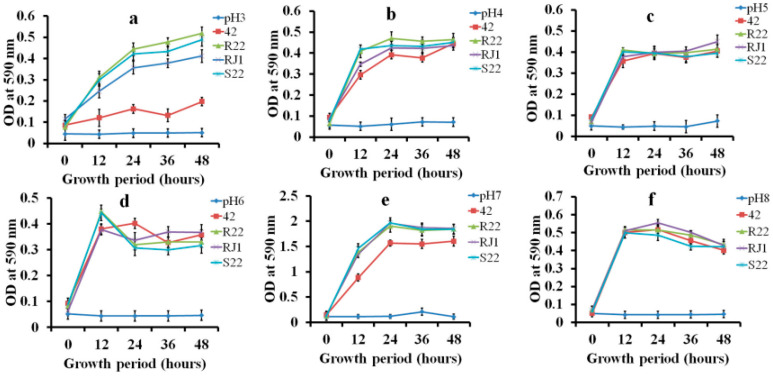
Growth ability of selected *Lactobacilli* strains (*L. plantarum* RJ1 and *P. pentosaceus* S22) in minimal carbohydrate media under micro-aerobic conditions. (a–f = pH-3, 4, 5, 6, 7, and 8 vs. time intervals of 0–48 h).

The growth of *L. plantarum* RJ1 and *P. pentosaceus* S22 on selective media with a low-carbohydrate source was not significantly different from growth in MRS broth. First, these two strains, *L. plantarum* RJ1 and *P. pentosaceus* S22, showed a stronger growth pattern than did other isolated strains (42 and R22). Figure 1 shows the optical density (OD_600_) values obtained for LAB strains in the minimal carbohydrate medium. These strains were capable of rapid cell proliferation in the low-glucose environment. Also, *L. plantarum* RJ1 and *P. pentosaceus* S22 showed the lowest pH values in the minimal low-carbohydrate MRS broth after 12 h incubation at 37 °C. Finally, these LAB isolates were selected as new silage inoculants and were named KCC-46 and KCC-47, respectively. In addition, the strains were identified as gram-positive and catalase-negative.

### 3.3. Molecular Characterization by 16S-rRNA Gene Sequencing 

The molecular markers of the LAB strains were identified using 16S-rRNA gene sequencing. The RJ11 strain shared more than 99% similarity with the species *L. plantarum*, whereas strain S22 was identified as *P. pentosaceus*, with a similarity of 97%. The closest phylogenetic neighbor of *L. plantarum* RJ1 was *L. plantarum* JCM 1149 (NR_117813) with an identity of 99%. Also, the *L. paraplantarum* DSM 10667 was next closest to RJ1 with an identity of 92%. Likewise, the *P. pentosaceus* S22 exhibited a distinct cluster together with *P. pentosaceus, P. parvulus, P. claussenii,* and *P. acidilactici,* with an identity of 95%. Furthermore, the authentic cultures were deposited in the Korean Agriculture Culture Collection (KACC), Korea; the strain reference numbers are KACC 92301P (*Pediococcus pentosaceus* KCC-46) and KACC 92302P (*Lactobacillus plantarum* KCC-47).

### 3.4. Biochemical Properties of Isolates

We evaluated the carbohydrate fermentation profiles of the isolates (RJ1 and S22) by the API 50 CHL micro-identification system. Table S1 shows the activity of *L. plantarum* RJ1 and *P. pentosaceus* S22 in 48 h of carbohydrate substrate fermentation. *L. plantarum* RJ1 showed a higher fermentation rate (at 24 h) than different industrially important carbohydrates. Next, the enzymatic activity pattern of the LAB isolates was tested with an API ZYM kit; the assessed results are presented in Table S2. Both *L. plantarum* RJ1 and *P. pentosaceus* S22 were positive for the production of 14 enzymes involved in synthesis of industry- and food-related products. In addition, the antibiotic sensitivity of the LAB strains against commercial antibiotics was evaluated using impregnated e-strips, as summarized in Table S3. *L. plantarum* RJ1 and *P. pentosaceus* S22 were strongly susceptible to azithromycin, gatifloxacin, neomycin, gentamycin, tetracycline, chloramphenicol, carbenicillin, and ciprofloxacin, and were moderately sensitive to norfloxacin, bacitracin, polymyxin-B, and clindamycin.

### 3.5. Antibacterial Activity of Isolates 

**Figure 2:**
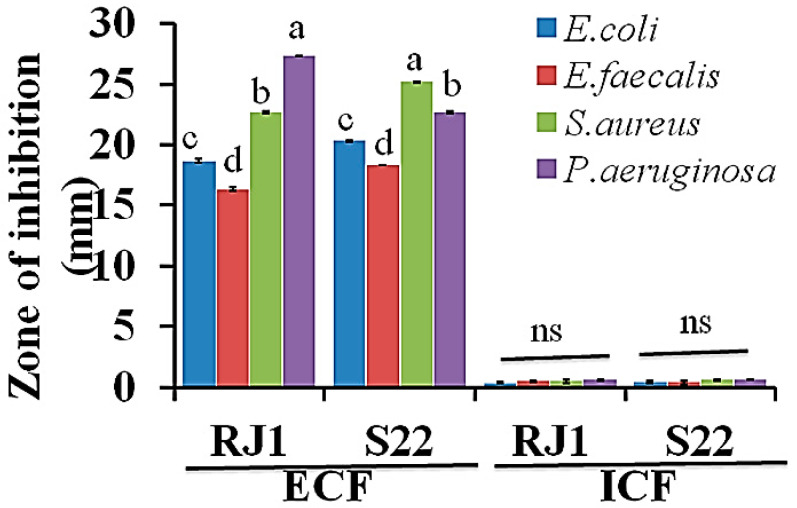
Antimicrobial activity of intra- and extracellular fluid of selected LAB strains against pathogenic bacteria according to the agar-well diffusion method. (ECF, extra cellular fluid; ICF, intracellular fluid from *L. plantarum* RJ1 and *P. pentosaceus* S22). The results are expressed as the mean values of three replicates (*n* = 3) and statistical data were analyzed by one-way analysis of variance (ANOVA) with Duncan’s multiple comparison test. Different letters (a, b, c) denote statistically significant differences between the ECF and ICF of LAB strains against pathogenic bacteria (*p* < 0.05).

Figure 2 shows the antibacterial activity of the ECF (cell free supernatants) and ICF (LAB cell fractions) of *L. plantarum* RJ1 and *P. pentosaceus* S22 against the tested pathogenic bacteria. The ECF of LAB cultures effectively inhibited the growth of harmful pathogens, such as *E. coli, S. aureus, E. faecalis, and P. aeruginosa*, and the inhibition zone was found to range between 16–25 mm. *L. plantarum* RJ1 displayed the highest antagonistic activity against *E. coli, S. aureus,* and *P. aeruginosa*. Similarly, *P. pentosaceus* S22 was noted to have an inhibition zone of 16 to 23 mm against *E. coli* and *E. faecalis,* respectively. Interestingly, the LAB strains grown in the minimal glucose (0.2%) medium, with glucose serving as a key source for LAB survival and organic acid production, showed similar antibacterial effects (data not shown). 

### 3.6. Antifungal Properties of Isolates 

**Figure 3:**
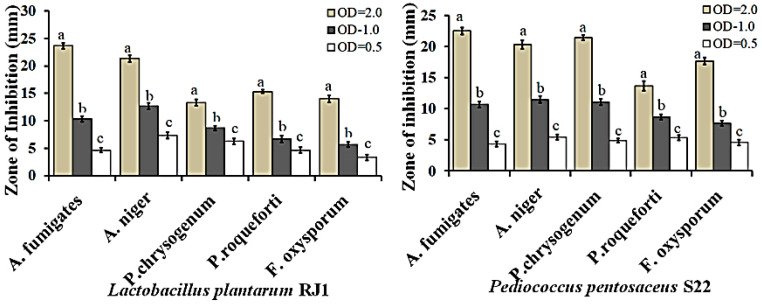
Antifungal activity of the selected LAB cultures at different concentrations (OD: 0.5, 1.0, and 2.0). Inhibitory activity of *L. plantarum* RJ1 and *P. pentosaceus* S22 was tested against five fungal targets. The results are expressed as the mean values of three replicates (*n* = 3) and data were analyzed by one-way ANOVA with Duncan’s multiple comparison test. Different letters (a, b, c) denote statistically significant differences between the various concentration of LAB strains (OD: 0.5, 1.0, and 2.0) against the selected fungal cultures (*p* < 0.05).

The antifungal activity of the two LAB strains against *P. chrysogenum, P. roqueforti, A. fumigatus, A. niger,* and *F. oxysporum* is shown in Figure 3. *L. plantarum* RJ1 and *P. pentosaceus* S22 strongly inhibited the growth of the filamentous fungus *A. fumigates* after 48 h. In particular, *L. plantarum* RJ1 moderately inhibited *P. chrysogenum* and *A. niger* growth after a 6-day incubation. In addition, *P. pentosaceus* S22 showed moderate inhibitory activity against *F. oxysporum* and *P. roqueforti* and low inhibitory activity against *P. chrysogenum* and *A. fumigatus*. The antifungal activity significantly differed between strains because of the varying concentrations of lactic acid, acetic acid, and other antifungal compounds (unknown) that reduce the growth of spoilage-promoting fungi.

### 3.7. Antioxidant Activity of Isolates 

**Figure 4:**
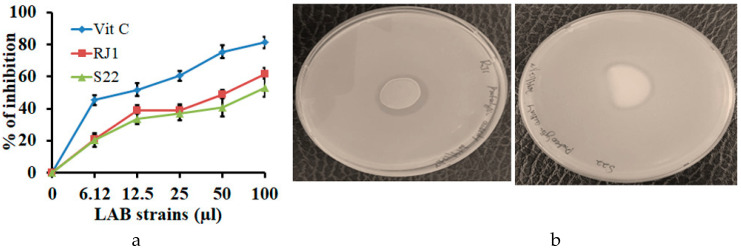
(**a**) DPPH free radical scavenging activity of RJ1 and S22 in vitro. The results are expressed as the mean values and standard error of three replicates (*n* = 3). (**b**) The proteolytic screening assay shows a clear zone around the LAB spot. (Left plate) *L. plantarum* RJ1 and (right plate) *P. pentosaceus* S22 on a skim milk agar plate.

The in vitro antioxidant activities of the selected LAB strains (RJ1 and S22) are presented in Figure 4a. The DPPH radical scavenging effect of the LAB strains ranged from 46 to 49%. *L. plantarum* RJ1 exhibited the highest scavenging ability, followed by *P. pentosaceus* S22, which showed the moderate scavenging activity (35–42%) compared to vitamin C (78%). 

### 3.8. Probiotic Characterization of LAB Isolates

#### 3.8.1. LAB Tolerance to Simulated Gastric and Intestinal Conditions

The tolerance of the isolates RJ1 and S22 to simulated gastric and intestinal fluid were examined at different time intervals. RJ1 and S22 could survive and exhibited slightly increased proliferation after a normal gastric phase incubation time, reaching survival rates above 93% (Table 1). The RJ1 strain had slightly more viable colonies (0.5–1 log CFU/mL) after 180 min than did S22. *P. pentosaceus* showed no significant changes in viability in the simulated gastric juice at pH 3.0. Food normally stays in the GI tract for about 2–4 h. Hence, survival of the isolates was assessed under acidic and bile salt conditions. RJ1 was able to survive in the simulated upper GIT stages, with a maximum survival rate of 80%, while *P. pentosaceus* S22 showed a significant reduction in cell viability in intestinal fluid (60%) after 24 h of incubation. Therefore, survival of the selected LAB strains in a simulated GI environment should be considered for further in vivo analysis and industrial applications.

#### 3.8.2. Bile Salt Tolerance of Isolates

Bile salt resistance is one of the most important selection principles for probiotic cultures. *L. plantarum* RJ1 and *P. pentosaceus* S22 exhibited different levels of resistance to bile salts after 12 h of exposure. In particular, the RJ1 strain was able to withstand a higher bile concentration (0.3% oxgall) with a tolerance of 84%. *P. pentosaceus* S22 had a slightly lower tolerance to bile salt with 81% at 12 h exposure (Table 2). 

### 3.9. Proteolytic Activity

The proteolytic activity assay revealed that RJ1 and S22 were able to produce a clear zone on skim milk agar, as shown in Figure 4b. In addition, the tested isolates secreted extracellular proteolytic enzymes that participate in hydrolysis and turn whitish smoky casein protein into colorless peptide fragments, thus appearing in a zone around the LAB. In addition, proteolysis occurred less in red clover silage, as indicated by a smaller percentage of peptide-N and free amino acid during the early stage of silage fermentation.

### 3.10. Cell Surface Hydrophobicity and Aggregation Properties of RJ1 and S22

The selected strains (RJ1 and S22) had a strong attraction for chloroform and ethyl acetate, and had moderate adherence to hexadecane. Also, both strains exhibited a greater affinity for acidic solvents (e.g., chloroform) and a lower affinity for basic solvents (e.g., ethyl acetate). The moderate adherence to a nonpolar solvent showed that the LAB isolates had an average percentage (40–50%) of cell-adhering hydrophobicity (Figure S1). The auto- and co-aggregation abilities of the isolates are presented in Figure S2. After 6 h of incubation, the highest percentages of aggregation were seen for *L. plantarum* (RJ1) and *P. pentosaceus* (24.37 and 27.98%, respectively). Also, the LAB strains revealed a higher co-aggregation ability with the intestinal pathogens *E. coli* and *S. aureus*.

### 3.11. Experimental Study of Silage Fermentation by LAB Isolates

Lower silage pH is good evidence of the quality of the fermentation process and indicates a higher concentration of organic acids in the final product. In this study, LAB isolate (RJ1, S22) inoculated legumes decreased the pH of the silage relative to non-treated silage. For alfalfa and crimson clover, silage pH decreased to 4.53 and 4.58 with the addition of LAB RJ1 and S22, respectively, while the non-inoculated control samples had a pH of 5.80 (Table 3 and Table 4). The differences in pH between legume silages may be attributable to differences in the water-soluble content of legumes, differences in the buffer capacity of the feed, or a combination of both. The pH of all control legume silage was above 5.8. The lower pH was dependent on the LAB population contained in the crimson clover and alfalfa (Table 3 and Table 4). Because silage fermentation and conservation can be particularly challenging in legume crops, the use of inoculants should be strongly considered to increase fermentation efficiency.

The LAB population was significantly greater in all legume silage samples treated with RJ1 and S22 at 180 days of fermentation than in non-treated legume silage samples. LAB-inoculated alfalfa and crimson clover showed greater organic acid production, mainly lactic acid (*p* < 0.05), which was five-fold higher than in the non-inoculated legume silage (Table 3 and Table 4). Other acids, such as butyric acid, were less concentrated in LAB-inoculated silage than in the control. In addition, butyric acid was not detected in LAB-inoculated silage, and the concentration of butyric acid was lower in all fermentation silage samples than in the controls, as shown in Table 3 and Table 4. Therefore, the LAB-inoculated legume silage had the highest lactic acid percentage on day 180 of fermentation. In this experiment, no yeast colonies were noted in RJ1- and S22-treated alfalfa samples; however, yeast was found in non-LAB-treated alfalfa silage (2.11 × 10^3^ CFU/mL). On the other hand, the crimson clover silage samples in the non-treated group contained more pink colonies (yeast) than in the LAB-treated group, at 1.52 × 10^3^ and 1.04 × 10^3^ CFU/mL, respectively. 

## 4. Discussion

LAB inoculation has a significant effect on silage quality and preservation. Silage production is a complex multistage biological process that involves various microbiological, biochemical, agronomical, soil biological, and engineering techniques [30,31]. Silage fermentation is determined by various factors, including anaerobic conditions in the ensiling, the amount of WSC, the epiphytic bacteria, the DM content of the forages, and the buffering capacity of the pre-ensiled plants. In this work, we isolated homo-fermentative LAB strains (*L. plantarum* RJ1, *P. pentosaceus* S22) that can grow in low-carbohydrate growth medium. The selected LAB strains showed faster growth rates than other LAB strains (including 42 and R22) and reduced the pH of the low-carbohydrate growth medium and MRS broth in micro-aerobic conditions (not shown). Natural cereal substrates can be used without the addition of carbohydrate supplements for growth characteristic analysis of probiotic strains, such as *L. fermentum*, *L. reuteri, L. plantarum*, and *L. acidophilus*; these strains attained a higher cell population in barley medium than in low-sugar malt medium [32]. However, Jin et al. (2012) [33] reported that lactose is utilized very slowly by LAB, and that fructose is a good alternative carbon source that produces faster growth and greater biomass. The lowered acidification rate in the minimal carbohydrate MRS medium (0.2% glucose) and the antibacterial activity of LAB strains were slow compared to that seen in high-glucose medium [34]. The phylogenetic tree analyses of the isolates fell within known groups of LAB, and they were related to particular species. The LAB that was characterized belonged to the genera *Lactobacillus*, with *L. plantarum* and *P. pentosaceus*. The phylogenetic grouping showed that isolates having similar sequences were assembled in the same cluster and considered as close relatives. The maximum composite likelihood method phylogenetic construction based on the 16S-rRNA genes clearly showed the *L. plantarum* and *P. pentosaceus* strains and their closest related species [35].

The antibacterial activity of LAB extracellular fluid and intracellular fluid was tested against *S. aureus, E. coli,* and *P. aeruginosa* pathogenic bacteria. In this study, we observed that variations in antibacterial activity of ICF and ECF, which are mainly due to the different compounds in the two, between the isolates might be caused by different antibacterial activity profiles against the selected pathogenic bacteria. Also, the extracellular fluid contains a higher amount of organic acids and protective substances, making it more effective in terms of antibacterial activity. Yi et al. (2016) [18] found that *L. coryniformis* exhibits potent antibacterial activity against *S. aureus*, and the isolated LAB acts against some selective pathogens alone. In the present study, the *L. plantarum* RJ1 and *P. pentosaceus* S22 strains showed concentration-dependent antibacterial effects against the tested pathogenic bacteria in vitro, and also showed a significantly increased inhibitory effect against *E. coli* and *S. aureus* in a concentration-dependent manner. 

The antimicrobial substances produced by LAB are important for use in the feed industry as safe and specific bio-preservatives [36,37]. The highest antifungal activity of RJ1 and S22 was seen in the MRS agar spot method after 48 h incubation. These results indicate that the RJ1 LAB strain had the highest antifungal effect against *A. fumigatus, A. niger,* and *P. roqueforti,* with a zone of inhibition of 24.5 ± 0.35, 21.33 ± 0.15, and 14.56 ± 0.33 mm, respectively. Also, the S22 LAB isolate has higher antifungal activity against *P. chrysogenum* and *A. fumigates* with a zone of inhibition of 23.5 ± 0.35 and 21.33 ± 0.15 mm, respectively. These results indicate that LAB strains produce antimicrobial agents such as organic acids that suppress fungal growth on MRS agar plates and in silage samples. Similarly, Guimaraes et al. (2018) [38] demonstrated that the antifungal activity of LAB is mainly linked with the production of organic acids, which are converted from sugar present in the growth medium. Previously, our team found that *L. plantarum* KCC-10 isolated from forages exhibited moderate activity against silage fungal pathogens. Pathogenic fungi, such as *A. niger, F. oxysporum,* and *P. chrysogenum,* were maximally sensitive to this effect; growth of *A. clavatus, A. fumigatus,* and *A. oryzae* was not suppressed by LAB [39]. Similarly, *P. pentosaceus* strains isolated from IRG forage exhibited strong inhibition against food spoilage-related pathogenic fungi [26]. In the present paper, we found that the selected inoculants, RJ1 and S22, moderately suppressed the growth of *F. oxysporum, A. fumigatus,* and *A. niger* fungi, which frequently damage silage. The protective activity of the strains results from the broad range of organic acid, mainly lactic acid, production and antifungal substances present in the growth medium.

The LAB isolates were strongly resistant to metronidazole, vancomycin, and lomefloxacin at 30 µg concentration. *Lactobacillus* sp. is generally sensitive to chloramphenicol, erythromycin, and tetracycline, but many LAB strains are susceptible to higher concentrations of streptomycin, and that susceptibility does not support intrinsic resistance to these commercial antibiotics [40]. Probiotic strains exhibited moderate antibiotic resistance to different groups of antibiotics. Also, Shazali et al. (2014) [41] reported that LAB was also susceptible to clindamycin, chloramphenicol, amoxicillin, ampicillin, penicillin, and erythromycin. However, this study indicates that the LAB strains have resistance to some common β-lactam antibiotics because of the absence of peptidoglycan in the LAB cell membrane.

Antioxidant substances donate electrons to free radicals to control cellular damage in animals, including humans. DPPH radical scavenging can be filtered by bacterial inoculum and can neutralize free radicals in the human system by up to 50%. However, when compared with synthetic antioxidants, the natural antioxidants have slightly lower activity [42], though they also have no adverse effects on human health and development. In addition, *L. plantarum* and *L. paracasei* cultures were shown to have a moderate antioxidant effect toward the DPPH free radical scavenger [2]. Collectively, our results indicate that rumen-isolated strains of *L. plantarum* RJ1 and *P. pentosaceus* S22 increase the antioxidant capacity of silage, and thus, may have practical uses in functional foods for the promotion of health and mitigation of disease.

LAB tolerance to gastric acidity (pH 3.0) is considered a primary functional requirement for probiotics. In this study, we found that the selected LAB strains, RJ1 and S22, were stable in viable numbers (8.59 and 8.28 log CFU/mL after 4 h of exposure to pH 3, respectively). Furthermore, studies in artificial intestinal fluid showed that the same strains had slightly reduced survival rates when incubated at pH 8 for 24 h (79% and 77%, respectively). Similarly, Kim et al. (2019) [3] studied the probiotic characteristics of LAB in the gastrointestinal tract. They found that, after 2 h incubation in an acidic environment (pH 2.5), the isolates exhibited a higher viability level, 97%, which indicates that the strain had good probiotic properties. Also, the cell viability counts of 14 LAB strains were found to be lower than 1 log CFU/mL after 4 h of exposure to pH 2.5 [43]. Therefore, the survival of LAB strains at a low pH can improve digestion and GI tract health. Lactic acid bacteria strains such as *L. delbrueckii* subsp. bulgaricus F17 showed the highest tolerance to bile salts with a short exposure time (0.48 h). In addition, this strain expressed a higher survival rate and better tolerance to simulated GI fluids [44]. The resistance to growth of LAB in this harsh environment was dependent on the concentration of bile acids [20,45]. So, bile concentration and incubation time play key roles in LAB survival and growth rate. Our results demonstrate that the selected strains possess good bile salt tolerance properties (in 0.3% oxgall). The LAB strains were able to survive under low pH and were slightly sensitive to oxgall (0.3%); however, these strains’ survival rate in bile salt is relatively high, so they could serve as potent probiotics. However, some selected LAB strains, e.g., *L. casei* R 465, were able to survive in low-pH environments and had improved proteolytic activity in fermented forage [46]. Protein hydrolysis occurs in ensiled forage through two major catabolic pathways. Also, plant enzymes, such as proteases, in silage additives play a main role in the conversion of free amino acids into ammonia (NH_3_) (i.e., oxidative deamination) [47,48]. 

Aggregation within the same strain of microorganisms (auto-aggregation) or between genetically different strains (co-aggregation) is vitally important in several ecological niches [24]. Probiotic bacteria play a significant role in preventing the surface colonization of pathogens. Therefore, LAB aggregation properties may contribute to one kind of defense mechanism for host for anti-pathogen activity. We found that the selected two LAB strains (RJ1 and S22) from the same rumen fluid were observed to have different percentages of the auto-aggregation level. The percentage level of LAB auto-aggregation may have been varied due to the intricate relationship among bacteria secreted factors, surface molecules, and membrane proteins. Also, the LAB expressing different bioactive metabolites that could be aggregation-promoting factors may further promote reducing pathogens via balancing the gut microbiota community and the co-aggregation mechanism [25].

LAB-based silage additives have been applied to promote the quality of feed in recent years. Generally, leguminous feed has a higher protein content and lower concentration of dry matter and WSC. For example, the average WSC of raw alfalfa is at most 40–60 g/kg DM, which is adequate for the fermentation of alfalfa silage. Also, the LAB population density was higher (5 log CFU/g fresh weight) than in non-treated silage, which promoted fermentation [49]. The findings of this research are consistent with previous reports, demonstrating that the natural fermentation process in forage crops and legume silage is controlled by *Lactobacillus* and *Pediococcus* species [50]. Anaerobic silage fermentation is a complex process that breaks down complex metabolites into organic acids. Fermentation highly depends on the presence of natural microbiota, the chemical composition of the forage crops, mainly WSC, and the presence of nitrogenous components [51]. Recently, Liu et al. (2019) [52] studied alfalfa silage and reported that LAB-inoculated silage has distinct shapes, colors, smells, and textures after 60 days of fermentation. The control silage (without inoculum) produced an unpleasant odor and seemed black and sticky. Also, the meta-analysis provides strong evidence that, in tropical grasses and alfalfa legumes, inoculation with lactic acid bacteria as a silage additive increases fermentation by increasing lactate accumulation and by reducing the pH and the growth of spoilage microbes such as *clostridia* and molds [53]. LAB isolates usually have positive effects on the fermentation of low-WSC crops such as *M. sativa, T. incarnatum*, and other legume silage. Among the isolates, *L. plantarum* showed a prominent ability to reduce the pH of alfalfa broth and to improve alfalfa silage quality [7].

The RJ1 and S22 strains displayed greater reductions in silage pH levels in alfalfa compared to red clover within 30 days of ensiling. Also, red clover reached a lower pH (4.21) than alfalfa did (4.32) during silage fermentation because it had more water-soluble sugars and a lower buffering capacity. The *L. Plantarum* RJ1 and *P. pentosaceus* S22 strains obtained from rumen fluid are confirmed as potential silage additives for alfalfa and crimson clover because they can survive, grow fast, and dominate the microbial environment in the silo. Inoculation with these efficient bacteriocinogenic strains rapidly stimulates fermentation and decreases pH within a month of ensiling. In addition, silage treated with homolactic strains have lower concentrations of acetic acid than silage treated with a commercial heterolactic inoculant [54]. Similarly, Guo et al. (2019) [16] found that prolonged storage time and treatment had a substantial effect on the composition of organic acid produced. In that study, with prolonged storage duration, the crude protein (CP) and nutrient profile were improved, but the amount of fermentable sugars and the LAB population were considerably decreased in all silage.

## 5. Conclusions

In conclusion, the isolated LAB strains, *L. plantarum* RJ1 and *P. pentosaceus* S22, from rumen fluid survived well in a low-carbohydrate medium, simulated gastrointestinal fluid, and high-bile salt environment. Also, these strains have a potent in vitro carbohydrate fermentation ability, as well as proteolytic and antagonistic activity against various pathogenic fungi and bacteria. *L. plantarum* RJ1 and *P. pentosaceus* S22 had a significant effect on organic acid production and LAB population in legume silage during the fermentation process. The addition of *L. plantarum* RJ1 and *P. pentosaceus* S22 elevated biochemical and fermentation characteristics and changed the quality of the high-moisture legume silage throughout the fermentation period. Also, the strains increased lactic acid production, decreased the pH quickly, and suppressed the growth of spoilage-related microorganisms. These animal-derived LAB strains could provide a new approach for the development of legume silage inoculants. The selected strains have great potential for use in legume silage fermentation at high moisture levels. Our findings must be confirmed in environments with different moisture levels, with a variety of storage periods, and in combination with other additives for high-quality legume silage production.

## Figures and Tables

**Table 1:** Viable LAB counts (log CFU/mL) in simulated gastric and intestinal fluid at pH 3 and pH 8 at different time intervals (Gastric fluid (GF): 0 and 4 h; Intestinal fluid (IF): 6, 12, and 24 h). Data represent mean ± SD (*n* = 3), and values within the same column with different superscript letters (a, b, c) are significantly different at *p* < 0.05. STD: standard LAB, NS: not significant at *p* < 0.05. (* Ref [20]).

Strains	Simulated Gastric Fluid (GF) Tolerance			Simulated Intestinal Fluid (IF) Tolerance			
	Simulated Gastric Fluid at pH3 (Log CFU/mL)		LAB Survival Rate (%)	Simulated Intestinal Fluid at pH8 (log CFU/mL)			LAB Survival Rate (%)
	0 h	4 h		6 h	12 h	24 h	
S22	8.52 ± 0.08 ^b^	8.28 ± 0.81 ^b^	97.18 ± 0.73 ^c^	7.85 ± 0.08 ^NS^	7.59 ± 0.01 ^b^	6.59 ± 0.06 ^a^	77.34 ± 1.24 ^b^
RJ1	8.76 ± 0.03 ^a^	8.59 ± 0.76 ^a^	98.05 ± 1.02 ^a^	7.83 ± 0.03 ^NS^	7.74 ± 0.02 ^a^	6.98± 0.06 ^b^	79.68 ± 0.88 ^a^
STD * (F17)	8.15 ± 0.41 ^c^	7.65 ± 0.12 ^c^	93.87 ± 1.43 ^b^	-	-	4.77± 0.31 ^c^	58.47 ± 3.86 ^c^

**Table 2:** Viable LAB counts (log CFU/mL) in simulated bile salt conditions at pH 5 for different time intervals (0, 12, and 24 h). Data represent mean ± SD (*n* = 3), and values within the same column with different superscript letters (a, b, c) are significantly different at *p* < 0.05. STD: standard LAB, NS: not significant at *p* < 0.05. (* Ref [20]).

Strains	Simulated Bile Salt Tolerance					
	MRS Broth (log CFU/mL)			MRS Broth + 0.3% Oxygall (log CFU/mL)		
	0 h	12 h	24 h	0 h	12 h	24 h
S22	1.19 ± 0.04 ^NS^	4.66 ± 0.02 ^b^	5.62 ± 0.02 ^c^	3.25 ± 0.04 ^NS^	2.28 ± 0.01 ^b^	2.08 ± 0.04 ^c^
RJ1	1.38 ± 0.07 ^NS^	5.92 ± 0.02 ^a^	7.88 ± 0.03 ^b^	3.23 ± 0.05 ^NS^	2.84 ± 0.07 ^a^	2.57 ± 0.03 ^b^
STD * (F17)	-	-	3.36 ± 0.01 ^a^	-	-	3.84 ± 0.01 ^a^

**Table 3:** Analysis of pH, microbial population, and organic acid concentrations of high-moisture LAB (*L. plantarum* RJ1 and *P. pentosaceus* S22)-inoculated and non-inoculated alfalfa silage on day 180 of incubation. Data represent mean values ± SD (*n* = 3), and superscript letters (a, b, c) within the same column are significantly different at *p* < 0.05. NS: not significant at *p* < 0.05.

Groups/Treatments	pH	Lactate (%)	Acetate (%)	Butyrate (%)	Total Bacteria (×10^7^CFU/g)
Control	5.56 ^b^	0.57 ^b^	0.16 ^b^	0.03 ^NS^	5.08 ^c^
*Lactobacillus plantarum* (RJ1)	4.53 ^a^	2.35 ^a^	0.24 ^a^	0.01 ^NS^	15.30 ^a^
*Pediococcus pentosaceus* (S22)	4.57 ^a^	2.24 ^a^	0.11 ^b^	0 ^NS^	12.50 ^b^

**Table 4:** Analysis of pH, microbial population, and organic acid concentrations of high-moisture LAB (*L. plantarum* RJ1 and *P. pentosaceus* S22)-inoculated and non-inoculated crimson clover silage on day 180 of incubation. Data represent mean values ± SD (*n* = 3), and superscript letters (a, b, c) within the same column are significantly different at *p* < 0.05. NS: not significant at *p* < 0.05.

Groups/Treatments	pH	Lactate (%)	Acetate (%)	Butyrate (%)	Total Bacteria (×10^7^CFU/g)
Control	5.83 ^a^	0.89 ^b^	0.38 ^a^	0.12 ^NS^	1.43 ^c^
*Lactobacillus plantarum* (RJ1)	4.58 ^b^	2.25 ^a^	0.30 ^a^	0 ^NS^	9.47 ^b^
*Pediococcus pentosaceus* (S22)	4.64 ^b^	1.75 ^a^	0.17 ^b^	0 ^NS^	16.71 ^a^

## Supplementary Materials

Supplementary materials can be found at https://www.mdpi.com/2076-2607/8/7/1044/s1; Figure S1: Assessment of cell-surface hydrophobicity of *L. plantarum* RJ1 and *P. pentosaceus* S22; Figure S2: The auto and co-aggregation abilities of RJ1 and S22 (SA-*Staphylococcus aureus*; EC-*E.coli* pathogens); Table S1: Carbohydrate-fermentation of RJ1 and S22 using API 50 CHL test kit; Table S2: Qualitative analysis of enzyme production of RJ1 and S22 using API-ZYM test kit; Table S3: Antibiotic resistance profile of *L. plantarum* RJ1 and *P. pentosaceus* S22 analyzed by the disc-diffusion method (S-susceptible; R-resistant; M-moderate).
