# Supplementary material for: Low-Carbohydrate Tolerant LAB Strains Identified from Rumen Fluid: Investigation of Probiotic Activity and Legume Silage Fermentation
Source: Microorganisms. 2020 Jul 14;8(7):1044. doi: 10.3390/microorganisms8071044 (PMC7409070; doi:10.3390/microorganisms8071044)
Supplement: Supplementary file 1 [file microorganisms-08-01044-s001.pdf]

Low-Carbohydrate Tolerant LAB Strains Identified from Rumen Fluid: Investigation of Probiotic Activity and Legume Silage Fermentation

Palaniselvam Kuppusamy [1,†], Dahye Kim [2,†], Soundharrajan Ilavenil [1], Hyung Soo Park [1], Jeong Sung Jung [1], Seung Hak Yang [1] and Ki Choon Choi [1,*]

[1] Grassland and Forages Division, National Institute of Animal Science, Rural Development Administration, Cheonan 31000, Republic of Korea; kpalaselvam@korea.kr (P.K.); ilavenil@korea.kr (S.I.); anpark69@korea.kr (H.S.P.); jjs3873@korea.kr (J.S.J.); y64h@korea.kr (S.H.Y.)

[2] Faculty of Biotechnology, College of Applied Life Science, Jeju National University, Jeju, 63243, Republic of Korea; pioioiq10@gmail.com

* Correspondence: choiwh@korea.kr; Tel.: +82-41-580-6752; Fax: +82-(041)-580-6779

† These authors contributed equally to this work.

Table S1. Carbohydrate-fermentation of RJ1 and S22 using API 50 CHL test kit.

S. No	Carbohydrates	RJ1	S22
1	Control	-	-
2	Glycerol	-	-
3	Erythritol	-	-
4	D-Arabinose	-	-
5	L.Arabinose	+	+
6	D-Ribose	+	+
7	D-Xylose	-	-
8	L-Xylose	-	-
9	D-Adonitol	-	-
10	Methyl-D-Xylopyraniside	-	-
11	D-Galactose	+	+
12	D-Glucose	+	+
13	D-Fructose	+	+
14	D-Mannose	+	+
15	L-Sorbose	+	-
16	L-Rhamnose	+	-
17	Dulcitol	-	-
18	Inositol	-	-
19	D-Mannitol	+	+
20	D-Sorbitol	+	-
21	Methyl-D-mannopyranoside	+	+
22	Methyl-D-glucopyranoside	+	-
23	N-Acetylglucosamine	+	+
24	Amygdalin	+	+
25	Arbutin	+	+
26	Esculin ferric citrate	+	+
27	Salicin	+	+
28	D-Celiobiose	+	+
29	D-Maltose	+	+
30	D-Lactose	+	+
31	D-Melibiose	+	+
32	D-Saccharose	+	+
33	D-Trehalose	+	-
34	Inulin	-	-
35	D-Melezitose	+	+
36	D-Raffinose	-	-
37	Amidon	-	-
38	Glycogen	-	-
39	Xylitol	-	-
40	Gentiobiose	+	-
41	D-Turanose	+	-
42	D-Lyxose	-	-
43	D-Tagatose	-	-
44	D-Fucose	-	-
45	L-Fucose	-	-
46	D-Arabitol	-	-

47	L-Arabitol	-	-
48	Potassium gluconate	+	+
49	Potassium gluconate 2-ketogluconate	-	-
50	Potassium gluconate 5-ketogluconate	-	-

+-presence; -absence.

Table S2. Qualitative analysis of enzyme production of RJ1 and S22 using API-ZYM test kit.

S. No	Extra Cellular Enzymes	RJ1	S22
1	Control	-	-
2	Alkaline phosphatase	+	+
3	Esterase (C4)	++	++
4	Esterase lipase (C8)	+++	++
5	Lipase (C14)	++	+++
6	Leucine arylamidase	++	+++
7	Valine arylamidase	++	+++
8	Cystine arylamidase	++	++
9	Trypsin	++	+
10	α-Chymotrypsin	+++	++
11	Acid phosphatase	+++	++
12	Naphthol-AS-biphosphohydrolase	+++	++
13	α -Galactosidase	+++	+++
14	β-Galactosidase	+++	+++
15	β -Glucuronidase	+++	+++
16	α -Glucosidase	+++	++
17	β -Glucosidase	+++	+++
18	N-acetyl-β-glucosaminidase	++	++
19	α -Mannosidase	-	+
20	α -Fucosidase	+	+

+++-high; ++- moderate; - no activity.

Table S3. Antibiotic resistance profile of *L. plantarum* RJ1 and *P. pentosaceus* S22 analyzed by the disc-diffusion method (S-susceptible; R-resistant; M-moderate).

S. No	Antibiotics	Disc Potency (mcg)	RJ1	S22
1	Lomefloxacin (LOM)	10	R	S
2	Norfloxacin (NX)	10	M	M
3	Azithromycin (AZM)	15	S	S
4	Vancomycin (VA)	30	R	R
5	Doxycycline hydrochloride		S	S
6	Co-Trimoxazole (COT)	25	S	S
7	Nitrofurantoin (NIT)	300	S	S
8	Gatifloxacin (GAT)	5	S	S
9	Carbenicillin (CB)	100	S	M
10	Cefoxitin (CX)	30	M	M
11	Clindamycin (CD)	2	M	S
12	Chloramphenicol (C)	30	S	S
13	Erythromycin (E)	15	S	M
14	Metronidazole (MT)	5	R	R
15	Penicillin (P)	10	M	R
16	Tetracycline (TE)	30	S	S

17	Bacitracin (B)	10	M	M
18	Polymyxin-B (PB)	300	M	R
19	Gentamycin (GEN)	10	S	M
20	Neomycin (N)	30	S	S

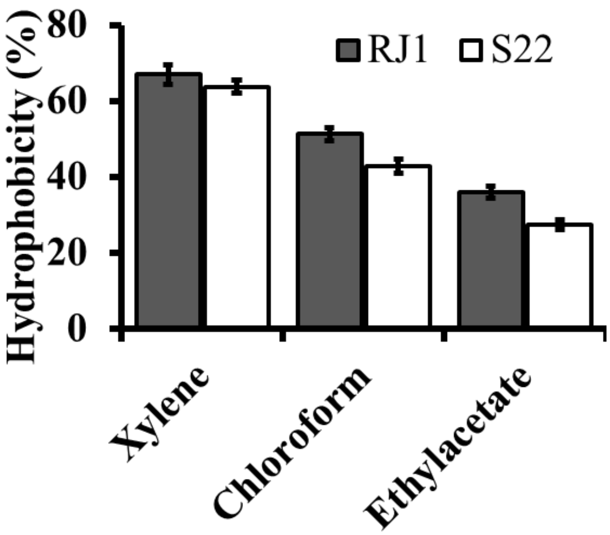

Figure S1. Assessment of cell-surface hydrophobicity of *L. plantarum* RJ1 and *P. pentosaceus* S22.

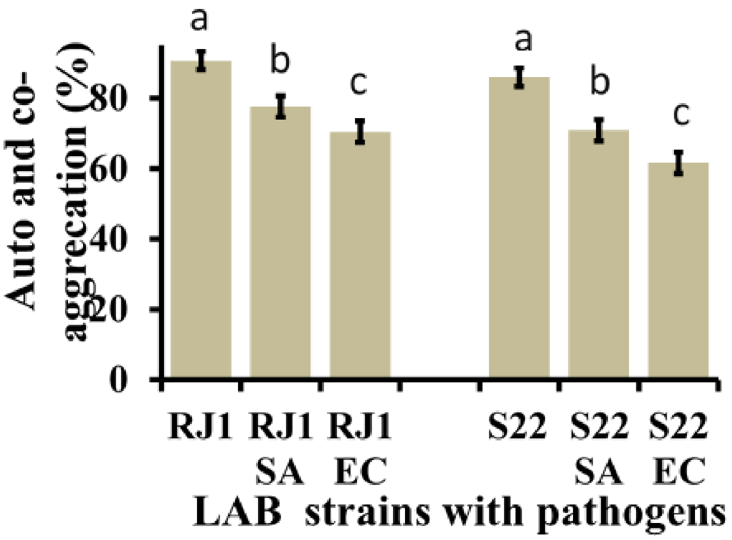

Figure S2. The auto and co-aggregation abilities of RJ1 and S22 (SA-*Staphylococcus aureus*; EC-*E.coli* pathogens).
